# Bayesian approach for predicting responses to therapy from high-dimensional time-course gene expression profiles

**DOI:** 10.1186/s12859-021-04052-4

**Published:** 2021-03-18

**Authors:** Arika Fukushima, Masahiro Sugimoto, Satoru Hiwa, Tomoyuki Hiroyasu

**Affiliations:** 1 Graduate School of Life and Medical Sciences, Doshisha University, Kyotanabe-shi, Kyoto 610-0321 Japan; 2 Research and Development Center for Minimally Invasive Therapies, Institute of Medical Science, Tokyo Medical University, Shinjuku, Tokyo 160-8402 Japan; 3 Institute for Advanced Biosciences, Keio University, Tsuruoka, Yamagata 997-0052 Japan; 4 Faculty of Life and Medical Sciences, Doshisha University, Kyotanabe-shi, Kyoto 610-0321 Japan

**Keywords:** Time-course data, Gene expression profiles, Bayesian, Prediction, Therapy response

## Abstract

**Background:**

Historical and updated information provided by time-course data collected during an entire treatment period proves to be more useful than information provided by single-point data. Accurate predictions made using time-course data on multiple biomarkers that indicate a patient’s response to therapy contribute positively to the decision-making process associated with designing effective treatment programs for various diseases. Therefore, the development of prediction methods incorporating time-course data on multiple markers is necessary.

**Results:**

We proposed new methods that may be used for prediction and gene selection via time-course gene expression profiles. Our prediction method consolidated multiple probabilities calculated using gene expression profiles collected over a series of time points to predict therapy response. Using two data sets collected from patients with hepatitis C virus (HCV) infection and multiple sclerosis (MS), we performed numerical experiments that predicted response to therapy and evaluated their accuracies. Our methods were more accurate than conventional methods and successfully selected genes, the functions of which were associated with the pathology of HCV infection and MS.

**Conclusions:**

The proposed method accurately predicted response to therapy using data at multiple time points. It showed higher accuracies at early time points compared to those of conventional methods. Furthermore, this method successfully selected genes that were directly associated with diseases.

**Supplementary Information:**

The online version contains supplementary material available at 10.1186/s12859-021-04052-4.

## Background

Predicting a patient’s response to therapy using various types of information is essential for designing systematic treatments [1, 2]. Hepatitis C virus (HCV) infection and multiple sclerosis (MS) are representative diseases showing individual variations that require personalized therapy. Systematic therapies utilizing pegylated interferon-alpha and ribavirin are recommended for the treatment of HCV infection [3]. However, only about half of all cases displayed a sustained response to this therapy [4]. Patients with HCV infection have reportedly exhibited serious neuropsychiatric side effects such as severe depression and psychosis [5]. Interferon-beta is the most widely used MS therapy to control disease symptoms [6]. However, this therapy did not prevent almost half of all patients from relapsing and even developing symptoms of brain disease, as observed in some cases [7]. To make appropriate decisions regarding therapeutic strategies, such as cancellation or fixation of long-term therapy, the therapeutic response associated with these diseases must be accurately predicted via time-course monitoring [8, 9]. Therefore, developing methods and markers that accurately predict individual therapeutic response is crucial for establishing successful long-term therapy.

Time-course gene expression profiling has advanced rapidly on account of time-course gene expression profiles collected from the same patient being more beneficial than those collected from the patient at a single time point [10, 11]. Methods that determine gene markers using time-course gene expression profiles are classified into two categories: statistical methods such as analysis of variance (ANOVA) [12, 13] and machine learning such as sparse modeling [14], decision trees [3], clustering [15, 16] and deep learning [17]. Many of these use standard problem settings to identify gene markers showing different time-course patterns between two groups, such as cases versus control. Detecting different patterns in time-course gene expression profiles is extremely beneficial for clarifying the biological processes involved. However, sometimes it may cause difficulties in predicting therapeutic response. For example, gene markers indicating a massive change between two late-term therapy groups may pose a challenge when it comes to making an accurate prediction for the first term. Conversely, gene markers that indicate significant early-term changes in treatment may make accurate late-term prediction difficult. Therefore, gene markers that accurately predict response to therapy at each observed time point are preferable for predictive purposes.

In predicting a long-term therapeutic response, prediction accuracy may be improved by incorporating patient information, which is repeatedly observed for a marker over time [18–20]. Rizopoulos et al. [19] and Li et al. [20] proposed a new method that dynamically updates predictive indicators as time points increase; they suggested that their method may improve prediction accuracy. However, these markers were not gene markers but aortic gradient levels [19] and brain imaging indices [20], which were also clarified as being useful by other studies. Therefore, the current study assumed that using more time points to profile a gene marker would lead to more accurate therapeutic response predictions.

Here, we propose a new prediction model and a gene selection method using time-course gene expression profiles. This method is based on the hypothesis that improving the accuracy of predictions requires more information obtained from gene markers at multiple time points. Therefore, our prediction model was designed to consolidate information from multiple time points, and our gene selection method was designed to identify gene subsets as markers that predict therapeutic responses more accurately with increasing time points. Time-course microarray datasets collected from HCV and MS patients were used to evaluate the proposed method. In this evaluation, three types of experiments were performed as follows: (1) comparison with our proposed method and the conventional method; (2) hypothesis verification; and (3) function analysis of the gene subset selected by the proposed method.

## Methods

Our proposed method was designed to predict therapeutic response using multiple time-point data that would expectedly yield a higher level of accuracy than a prediction based on single-point data. Our method is termed ‘the consolidating probabilities of multiple time points (CPMTP) method’. CPMTP consists of prediction procedure (CPMTPp) and gene selection procedure (CPMTPg). Section 2.1 introduced the theory of CPMTPp and CPMTPg. Section 2.2 described the numerical experiments.

### Theory

This section described CPMTPp and CPMTPg in the Sections 2.2.1 and 2.1.2, respectively. Briefly, CPMTPp is the procedure for predicting therapy response using a model. CPMTPg is the procedure for selecting genes.

#### Concept of CPMTPp

**Fig. 1:**
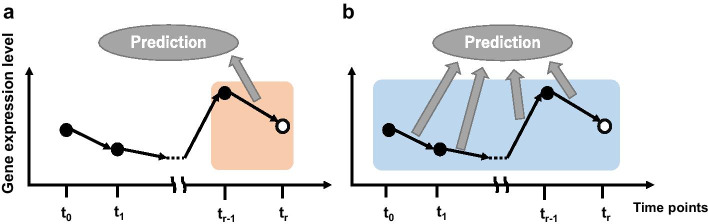
The concept of predicting therapeutic response using the conventional method and our proposed method; **a** The conventional method used single gene expression levels or differential gene expression levels between two time points. **b** Our proposed method used time-course gene expression profiles according to consolidated probabilities of multiple time points

The CPMTPp design was based on the hypothesis that predictive accuracy is improved by consolidating information on the states of a patient at multiple time points. The general problem setting for the prediction in which the response at future time point “${\mathrm{T}}_{final}$” was estimated as either “sensitive” or “not sensitive” using gene expression profiles is shown (Fig. 1). “Sensitive” meant that the patients responded well to therapy and recovered from the disease, and “not sensitive” meant that patients could not recover from the disease with the therapy. The time points corresponding to gene expression profiles used for prediction by CPMTPp and conventional methods were different. In this paper, the time points used in each method are termed ‘checkpoint (CP).’ The CP of conventional methods was a single time point “${t}_{r} (r=0,\dots ,R)$” or the difference between the one-time point “${t}_{r} ({t}_{r}\le {\mathrm{T}}_{final})$” and the previous time point “$t-1$” (Fig. 1a); it was, thus, mostly confined to two-time points. Meanwhile, the CPMTPp used the gene expression profiles corresponding to the first time point “$t0$” to a time point “$t$” (Fig. 1b); here, more than two time points were used for predictive purposes. In this manner, the hypothesis was implemented using CPMTPp by consolidating the probabilities of therapeutic response using gene expression profiles collected from multiple time points.

**Fig. 2:**
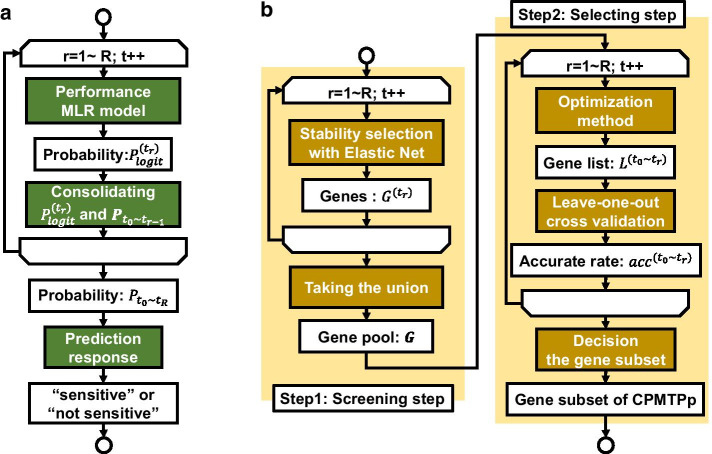
The flow of CPMTP; **a** CPMTPp was based on the hypothesis that information at multiple time points improves predicted accuracy. **b** CPMTPg select the gene subset used by the CPMTPp model

CPMTPp was used to calculate one probability of therapeutic response using time-course microarray data (Fig. 2a). Firstly, CPMTPp was used to calculate a probability using the gene expression profile at a time point, “${t}_{r}$”. Secondly, the probability at “${t}_{r}$” and the prior probability from “${t}_{0}$” to “${t}_{r-1}$” was consolidated to calculate a more accurate probability. By repeating these two steps until “$\mathrm{r}=R$,” the probabilities at multiple time points were aggregated into one probability (where “T” was the final time point that can be used for prediction).

Similar to the Bayesian model [21, 22] and neural network [23], multiple logistic regression (MLR) models have been widely used to predict the response to therapy based on probability. This probability did not present a p-value in statistical tests but present how likely the patient is likely sensitive (or not sensitive). The probability at the first step, “${\mathrm{P}}_{logit}^{({t}_{r})},$” was calculated through MLR, like Eq. 1, using the difference of gene expression profile between “${t}_{r}$” and “${t}_{r-1}$” (Eq. 2). However, these models used single or two time points to calculate the probability and did not use time-course data.

1 $${\mathrm{P}}_{logit}^{({t}_{r})}=\frac{1}{1+{e}^{-({w}_{0}^{({t}_{r})}+{w}_{1}^{({t}_{r})}*{d}_{1}^{({t}_{r})}+\dots +{w}_{l}^{({t}_{r})}*{d}_{l}^{({t}_{r})})}}$$

${d}_{j}^{({t}_{r})} (j=1,\dots , l)$: different expression levels of the jth gene between two-time points “${t}_{r}$” and “${t}_{r-1}$”. $"l$” is the number of genes in a gene subset of logistic regression. The gene subset was selected from all the genes collected by microarray using CPMTPg.

${w}_{j}^{(t)} (j=0,\dots ,l)$: the weight of jth gene as a feature in a gene subset. ${w}_{0}^{({t}_{r})}$ is a constant term at time point “${t}_{r}$.”

2 $${d}_{j}^{({t}_{r})}={x}_{j}^{({t}_{r})}-{x}_{j}^{({t}_{r-1})}$$

${x}_{j}^{({t}_{r})}$: jth gene expression levels at time point “${t}_{r}$.” ${x}_{j}^{({t}_{r-1})}$ is the jth gene expression level at time point “${t}_{r-1}$”.

In CPMTPp, the Bayesian theory was used to consolidate probabilities based on time-course data [7]. The probability, “${\mathrm{P}}_{{t}_{0}\sim {t}_{1}},$” was calculated by combining the probability at time point “${\mathrm{P}}_{logit}^{({t}_{r})}$” and the probability at previous time points “${\mathrm{P}}_{{t}_{0}\sim {t}_{r-1}}$” (Eq. 3). As the previous time point did not exist ($r=1$), $"{\mathrm{P}}_{{t}_{0}\sim {t}_{1}}={\mathrm{P}}_{logit}^{({t}_{1})}"$ was defined. ${\mathrm{P}}_{{t}_{0}\sim {t}_{r}} 0.5$ and ${\mathrm{P}}_{{t}_{0}\sim {t}_{r}}<0.5$ indicate sensitive and not sensitive responses, respectively (Eq. 4). From the above, CPMTPp could be used to predict response to therapy based on gene expression profiles at multiple time points.

3 $${\mathrm{P}}_{{t}_{0}\sim {t}_{r}}=\frac{{\mathrm{P}}_{logit}^{({t}_{r})}*{\mathrm{P}}_{{t}_{0}\sim {t}_{r-1}}}{{\mathrm{P}}_{logit}^{({t}_{r})}*{\mathrm{P}}_{{t}_{0}\sim {t}_{r-1}}+(1-{\mathrm{P}}_{logit}^{({t}_{r})})*(1-{\mathrm{P}}_{{t}_{0}\sim {t}_{r-1}})}$$

${\mathrm{P}}_{logit}^{({t}_{r})} \left({t}_{r}={t}_{0},\dots ,{t}_{R}\right):$ A probability of sensitive (or not sensitive) response to therapy using gene expression profile at time point “${t}_{r}$”.

4 $$Predicted\;therapy\;response: = \left\{ {\begin{array}{*{20}l} {sensitive} & {\left( {{\text{P}}_{{t_{0} \sim\,t_{r} }} \ge 0.5} \right)} \\ {not\;sensitive} & {({\text{P}}_{{t_{0} \sim\,t_{r} }} < 0.5)} \\ \end{array} } \right.$$

${\mathrm{P}}_{{t}_{0}\sim {t}_{r}} \left({t}_{r}={t}_{0},\dots ,{t}_{R}\right):$ A probability of sensitive (or not sensitive) response to therapy using gene expression profile at time points “${t}_{0}\sim {t}_{R}$”.

#### Algorithm of CPMTPg

CPMTPg were used to select the gene subset of CPMTPp best suited for accurate prediction using time-course microarray data. CPMTPg was used to decide the gene subset by optimizing the fitness function based on the probability “${\mathrm{P}}_{{t}_{0}\sim {t}_{r}}$” used in CPMTPp. This function was designed with negative penalties for incorrect predictions. The CPMTPg flowchart, which consists of gene screening (step 1) and deciding on a gene subset (step 2), is shown (Fig. 2b).

Step 1: Elastic net with stability selection eliminated genes with low impact on therapy responses, yielding a gene pool.

Step 2: The gene subset was selected from the gene pool via an optimization method.

Here, the gene expression profiles were composed as a data matrix (“$l$” genes × “$N$” subjects × “$R$” time points). Each subject was labeled as “sensitive” or “not sensitive” based on therapy responses.

**Step 1: Screening step**

Gene selection based on microarray data frequently suffers from the “$n \gg p$ problem,” i.e., a large number of genes ($p$) compared to the small number of samples ($n$) [24]. Gene selection using univariate analyses causes an α-error by independent multiple tests. These p-values should be corrected via adjusting using methods such as the Bonferroni correction [25], Holm method, or Dunnett’s method [13]. However, sparse modeling enables the selection of genes without p-values.

The sparse modeling solved the “$n \gg p$ problem” by considering a condition where only a few genes affect the phenomenon under focus [14, 15, 26]. We employed “Elastic net,” a sparse modeling method [27]. Elastic net selects a subset effectively from features with high multicollinearity. To eliminate genes with minimal impact on therapeutic responses, an Elastic net was applied to gene expression data at each time point.

Elastic net was used to select genes with non-zero weights, ${w}_{j}^{({t}_{r})},$ which are used for an MLR model according to Eq. 5. The genes with zero weights indicate that these genes were not selected as a gene subset for the MLR model. The Elastic net equation added regularized terms (the second and third terms of Eq. 5) to the general loss function, such as the least-squares method (the first term of Eq. 5) to optimize the weights. The second term prevents multicollinearity, and the third term selected features. Because the genes selected by Elastic net depended on the value of lambda in Eq. 5, deciding lambda was important to predict the response to therapy accurately.

5 $$\mathop {{\text{argmin}}}\limits_{{{\varvec{w}}^{\left( t \right)} }} J\left( {{\varvec{X}}^{{\left( {t_{r} } \right)}} ,{\varvec{y}}} \right) + \lambda \left( {\mathop \sum \limits_{j = 1}^{l} \left( {1 - \alpha } \right)\frac{1}{2}\left| {w_{j}^{{\left( {t_{r} } \right)}} } \right|^{2} + \mathop \sum \limits_{j = 1}^{l} \alpha \left| {w_{j}^{{\left( {t_{r} } \right)}} } \right|} \right)$$

${{\varvec{w}}}^{({t}_{r})}={({w}_{1}^{\left({t}_{r}\right)},\dots ,{w}_{l}^{\left({t}_{r}\right)})}^{\mathrm{\top }}$: the weights of a logistic regression model at a time point”${t}_{r}$” in Eq. 4. ${{\varvec{X}}}^{({t}_{r})}=\left({{\varvec{x}}}_{1}^{\left({t}_{r}\right)},\dots ,{{\varvec{x}}}_{l}^{\left({t}_{r}\right)}\right); {{\varvec{x}}}_{j}^{\left({t}_{r}\right)}={\left({x}_{j}^{\left(1,{t}_{r}\right)},\dots ,{x}_{j}^{\left(N, {t}_{r}\right)}\right)}^{\mathrm{\top }}:$ the difference in jth gene expression levels at a time point between “${t}_{r}$” and “${t}_{r-1}$”. ${\varvec{y}}=\left({y}^{1},\dots ,{y}^{N}\right);{y}^{i} \in \{\mathrm{0,1}\}$: Therapy response of ith patient at time point$t$. If${y}^{i}=1$, the therapy response presents “sensitive” If not, the therapy response presents “not sensitive.”$J\left({{\varvec{X}}}^{\left({t}_{r}\right)},{\varvec{y}}\right)$: Loss function of a logistic regression model using ${{\varvec{X}}}^{\left({t}_{r}\right)}$ and ${\varvec{y}}$.$\lambda$: A hyper-parameter that represents the weight of the regularized terms in Elastic Net.$\mathrm{\alpha }(0<\mathrm{ \alpha }< 1)$: A hyper-parameter that decides the assignment of the second and third terms.

Stability selection was used to reduce the effect of lambda on feature selection [28, 29]. Stability selection performed Elastic net many times with various lambda values to sub-sample sets via random sampling. A gene pool at each time point (${G}^{({t}_{r})}$ in Fig. 2b) was created based on the selected rate in repeated times at a lambda value. At step 1, the gene list (${\varvec{G}}$ in Fig. 2b) consisted of genes belonging to gene pools at any of the time points “${{\{G}^{({t}_{0})},\dots ,G}^{({t}_{R})}\}$”. In this step, some genes that affected prediction at each time point could be selected from a huge number of genes in the microarray data.

**Step 2: Selecting a gene subset**

In step 2 of CPMTPg, the gene subset for the CPMTPp model was selected from the gene pool “$G$” via optimization. For CPMTPg, the gene list (${L}^{({t}_{0}\sim {t}_{r})}$ in Fig. 2b) was created by combinatorial optimization method.

This step was performed as follows:

(i) The gene list (${L}^{({t}_{0}\sim {t}_{r})}$ in Fig. 2b) was selected from gene expression profiles at time points from “${t}_{0}$” to “${t}_{r}$” via the optimization method.
(ii) The subjects in the gene expression data were separated into two blocks.
(iii) The CPMTPp model was constructed based on one block of data using the gene list ${L}^{({t}_{0}\sim {t}_{r})}$.
(iv) The accurate rate of the model (${acc}^{({t}_{0}\sim {t}_{r})}$ in Fig. 2b) was calculated using the other block of data.
(v) (ii) and (iv) were repeated for “$r=$ R.”
(vi) The gene list showing the best accuracy rate was determined as the gene subset of CPMTPp.

The fitness function of the optimization method was designed via probability consolidated at multiple time points (Eq. 6). Equation 6 used the probability, “${P}_{{t}_{0}\sim {t}_{r}}^{\left(s\right)}$,” and the number of accurate predictions in patients, “${N}_{true}$,” as a reward, and the probability, “${P}_{{t}_{0}\sim {t}_{r}}^{\left(q\right)}$,” and the number of patients with no incorrect predictions, “${N}_{False}$,” as a penalty. The absolute value of the difference between probabilities and “$0.5$” ($\left|{P}_{{t}_{0}\sim {t}_{r}}^{\left(s\right)}-0.5\right|$ and $\left|{P}_{{t}_{0}\sim {t}_{r}}^{\left(q\right)}-0.5\right|$ in Eq. 6) presented a confidence level of the predicted therapy response. If probabilities ${P}_{{t}_{0}\sim {t}_{r}}^{\left(s\right)}$ and ${P}_{{t}_{0}\sim {t}_{r}}^{\left(q\right)}$ were closer to “0” or “1”, respectively, these values were higher. However, if these probabilities were closer to “0.5”, these values were lower. Therefore, the first and second terms of Eq. 6 are the mean values of the confidence levels of accurate and incorrect predicted therapeutic responses, respectively. The optimization method selects the gene subsets with a CPMTPp that can accurately predict and display high confidence levels for the predicted response to therapy by maximizing the fitness function of Eq. 6.

6 $$Fitness := \frac{1}{{N_{true} }}\mathop \sum \limits_{s = 1}^{{N_{true} }} \left| {P_{{t_{0} \sim\,t_{r} }}^{\left( s \right)} - 0.5} \right| - \frac{1}{{N_{false} }}\mathop \sum \limits_{q = 1}^{{N_{false} }} \left| {P_{{t_{0} \sim\,t_{r} }}^{\left( q \right)} - 0.5} \right|$$

${N}_{true}$: Number of patients in whom the actual therapy response equaled the predicted one. ${N}_{false}$: Number of patients in whom the actual therapy response did not equal the predicted one. ${P}_{{t}_{0}\sim {t}_{r}}^{\left(s\right)} (\mathrm{s}=1,\dots ,{N}_{true})$: Probability of *s*th patients that the actual therapy response equaled the predicted one. ${P}_{{t}_{0}\sim {t}_{r}}^{\left(q\right)} (\mathrm{q}=1,\dots , {N}_{false})$: Probability of *q*th patients that the actual therapy response did not equal the predicted one.

To determine the gene subset of CPMTPp from gene lists “{${L}^{({t}_{0}\sim {t}_{1})},\ldots,{L}^{({t}_{0}\sim {t}_{R})}$}”, the accurate rate “${acc}^{({t}_{0}\sim {t}_{r})}$” was calculated by the (ii)–(iv) flows. In these flows, leave-one-out cross-validation was used. The number of patients in two blocks of data by this cross-validation was 1 for evaluation and “$N-1$” for the construction of the model. The accuracy rate was shown as the proportion of patients whose predicted therapeutic responses were accurate for evaluating the cross-validation. CPMTPg made it possible to construct a CPMTPp model that enabled accurate prediction at multiple time points using the gene list with the highest accuracy rate as the gene subset in CPMTPp.

Note that this step used Ridge as an optimization method for weights $"{w}^{({t}_{r})}"$ in an MLR model (Eq. 1) to calculate “${P}_{{t}_{0}\sim {t}_{r}}^{\left(s\right)}$” and “${P}_{{t}_{0}\sim {t}_{r}}^{\left(q\right)}$” in Eq. 3. Ridge does not select genes and constructs the model to avoid multicollinearity. At “$\alpha =0$” of Eq. 5, this equation is not Elastic net, but Ridge.

### Numeric experiments

Three experiments were performed: (1) comparison with CPMTP (CPMTPp + CPMTPg) and a conventional method, (2) verification of our hypothesis, and (3) analysis of the gene subset selected by CPMTPg. This section describes the material, preprocessing, evaluation method, parameters, and implementation.

#### Material and preprocessing

Two sets of time-course microarray data were used for this evaluation. One dataset was collected from HCV patients treated with antiviral therapies, peginterferon and ribavirin (HCV dataset) [30]. The other was collected from MS patients treated with interferon-β (MS dataset) [31]. These datasets (GSE7123 and GSE24427) were opened on the GEO website [32].

The details of these datasets are shown (Table 1). The number of time points in the HCV dataset was six (${t}_{0}$ to ${t}_{5}$), and the difference in CP between CPMTPp and a conventional method is shown (Table 2). There were five MS datasets (${t}_{0}$ to ${t}_{4}$), and the difference in CP between CPMTP and a conventional method is shown (Table 3). Gene expression profiles were collected using the Affymetrix Human Genome U133A Array from peripheral blood mononuclear cells of patients, where the patients used for this evaluation were limited to those who could provide these at all time points.

**Table 1:** Summary of time-course gene expression profiles collected from HCV and MS patients

The number of data	HCV data	MS data
Genes	13,513	13,513
Time-points	${t}_{0}$(0 day), ${t}_{1}$(1 day), ${t}_{2}$(2 days)${t}_{3}$(7 days), ${t}_{4}$(14 days), ${t}_{5}$(28 days)	${t}_{0}$(first), ${t}_{1}$ (second), ${t}_{2}$(1 month), ${t}_{3}$(12 months), ${t}_{4}$(24 months)
Sensitive/not sensitive responders	Sensitive: 36 Not sensitive: 22	Sensitive: 16 Not sensitive: 9
Sensitive/not sensitive responders for stability selection	Sensitive: 28 Not sensitive: 17	Sensitive: 12 Not sensitive: 7
Sensitive/not sensitive responders for *k*-fold cross-validation	Sensitive: 12 Not sensitive: 7–8	Sensitive: 10–11 Not sensitive: 6

The number of genes of both HCV and MS is 13513, but the types of genes are different from them. In this paper, symbols of time points were presented as “${t}_{0}$”, “${t}_{1}$”, “${t}_{2}$” etc. The number of sensitive/not sensitive responders for the k-fold cross-validation varies by block

**Table 2:** The difference in CPs between the conventional method and CPMTP in the HCV dataset

Time-point	CP1	CP2	CP3	CP4	CP5
${t}_{0}$	# *	*	*	*	*
${t}_{1}$	# *	# *	*	*	*
${t}_{2}$		# *	# *	*	*
${t}_{3}$			# *	# *	*
${t}_{4}$				# *	# *
${t}_{5}$					# *

“#” and “*” were the time points of microarray data used by the conventional method and CPMTP, respectively

**Table 3:** The difference in CPs between the conventional method and CPMTP in the MS dataset

Time point		CP1	CP2	CP3	CP4
${t}_{0}$		# *	*	*	*
${t}_{1}$		# *	# *	*	*
${t}_{2}$			# *	# *	*
${t}_{3}$				# *	# *
${t}_{4}$					# *

“#” and “*” were the time points of microarray data used by the conventional method and CPMTP, respectively

Three steps were performed to preprocess gene expression data. Several probes were removed from the two datasets. As the probes had duplicate gene symbols in one dataset, one probe was selected by comparing median gene expression levels, and the other probes were removed. Probes with a gene symbol indicating a non-coding region or no gene symbol were also removed. Subsequently, log_2_ transformation and quantile normalization were performed on each dataset.

#### Conventional method

The MLR model was used as the prediction model for the conventional method. The features of the MLR model were based on the difference of gene expression profiles between “$t$” and “$t-1$”. The CPs of the MLR model for the HCV and MS datasets are shown in Tables 2 and 3, respectively.

Next, maSigPro was used for gene selection in the conventional method, which is frequently used for time-course microarray data analysis [25, 33, 34]. This method selects the gene subset which shows a time-course difference in gene expression profiles between two groups via p-values of a statistical test with the significant level of “${s}_{maSigPro}$”. This p-value was associated with F-statistic and was corrected by the linear step-up false discovery rate procedure. When the number of genes selected by maSigPro was over “${l}_{max}$”, “${l}_{max}$” genes were selected in ascending order of p-values.

#### Evaluation method

To compare CPMTPp + CPMTPg and MLR + maSigPro as the conventional method, the area under the curve (AUC) and accuracy were calculated using HCV and MS datasets. For this, k-fold cross-validation was performed. This method splits patients in the dataset into “$k$” blocks. The k − 1 blocks were used for the model training, and the remaining 1 block was used for evaluation. This procedure was repeated k times, and all data were used for evaluation at one time.

The receiver operating characteristic curves (ROCs) for each CP, which were calculated based on probabilities of CPMTPp + CPMTPg and MLR + maSigPro that were obtained via k-fold cross-validation, is depicted. The AUCs were calculated using these ROC curves. The difference between AUCs corresponding to CPMTPp + CPMTPg and MLR + maSigPro at each CP were compared using the DeLong test with significance levels “${s}_{AUC}$”.

To compare with CPMTP and previous studies based on therapy responses estimated via k-fold cross-validation, the accuracies of CPMTPp + CPMTPg and MLR + maSigPro were calculated. The accuracies were calculated for each CP and each block for evaluation in k-fold cross-validation. Based on the mean, maximum, and minimum values of these accuracies, CPMTPp + CPMTPg and MLR + maSigPro were compared.

In CPMTPp, it was assumed that the accuracy of the prediction model was improved as time points increased. The accuracies of the CPMTPp and MLR models were compared to verify this hypothesis. The gene selection methods of these models were CPMTPg. The mean, maximum, and minimum values of accuracies in CPMTPp + CPMTPg and MLR + CPMTPg were calculated using k-fold cross-validation using HCV and MS datasets.

The gene subset selected by CPMTPg was analyzed by ontology to research the function of genes in the biological process. DAVID [35] was used as an ontology analysis tool. Common terms (GO terms) that were associated with the genes of CPMTPg were decided using DAVID based on p-values below the significance level “${s}_{DAVID}$”. This p-value was a modified Fisher exact p-value. Also, previous studies were investigated using GO terms as keywords.

#### Parameters and implementation

The therapeutic responses of patients were decided at the final time point of the datasets. The final time point in the HCV dataset was 28 days (“${t}_{5}$”) after the first therapy. In the HCV dataset, the types of therapy responses were categorized as "marked,” “intermediate,” and “poor”. A “marked” response was defined as “$decreasing \, RNA \, levels \, of \, HCV > 3.5 \,log10 \;\mathrm{IU}/\mathrm{ml}$” or no detected levels on day 28. An “intermediate” response was defined as “$decreasing \, 1.4 \le RNA \, levels \, of \, HCV \le 3.5 \, log10\; \mathrm{IU}/\mathrm{ml}$” on day 28. A “poor” response was defined as “$decreasing \, RNA \, levels \, of \, HCV <1.4 \, log10 \;\mathrm{IU}/\mathrm{ml}$” on day 28. However, for this evaluation, the "marked" and "intermediate" responses were considered as “good” results, as in previous studies [3]. Responses to therapy based on the MS dataset were decided by the occurrence of relapse up to 24 months (“${t}_{4}$”) after first therapy, and they were considered “good” or “poor”. In this paper, a “good” response was treated as “sensitive,” and a “poor” response was treated as “not sensitive.”

The parameters of Step 1 in the CPMTPg are as follows. Stability selection was repeated 100 times. Stability selection selected 80% of patients from each “sensitive” and “not sensitive” category as the sub-sample set (Table 1). Lambda values corresponding to repetition were created based on the exponential function from ${log10}^{-3}$ to $log3$. The alpha value of Elastic net in Stability selection was “$0.5$”.

The parameters of Step 2 in the CPMTPg were as follows. A genetic algorithm (GA) was utilized as the optimization method. GA is a heuristic optimization method that has been frequently utilized as a gene selection method for microarray data [24, 36, 37]. GA repeats single-point crossover, ranking selection, and mutation at each generation. The number of generations was 50, and the population size of each generation was “20.” The phenotype of GA is a binary presented as either to select or not select candidate genes. Note that the maximum number of genes selected for each population was 10 and that the population for the first generation was created by random sampling. To create the next generation, a single-point crossover was generated twice in the population, and mutation was performed on 20% of the population. The mutation reversed the select or not select process at a randomly chosen locus in the population. Based on the fitness values in Eq. 6, ranking selection identified the top 40% and the bottom 10% of the total population as the next generation.

The parameters of the numeric experiment are as follows: “k = 3” in k-fold cross-validation, and the rate of patients whose therapy responses were “sensitive” or “not sensitive” was the same for all blocks (Table 1). The parameters of maSigpro were “${s}_{maSigPro}=0.05$” and “${l}_{max}=10$ The significance level of the DeLong test and DAVID were “${s}_{AUC}=0.05$” and “${s}_{DAVID}=0.05,$” respectively.

The implementation language was R-Language (ver. 3.6.0). Quantile normalization, Elastic net, and maSigPro were used by limma (ver. 3.40.6), glmnet (ver. 2.0-18), and maSigPro (ver. 1.56.0) packages, respectively. Stability selection and GA were implemented by the authors. The source codes used in this paper will be made available upon request. The pseudo code of CPMTP was added in Additional file 1: Figure S1, Additional file 2: Figure S2, and Additional file 3: Figure S3.

## Results

A threefold cross-validation was performed for each HCV and MS dataset. In MLR + maSigPro and CPMTPp + CPMTPg, AUCs, as well as mean maximum and minimum values of accuracies, were calculated based on the results of cross-validation. The mean, maximum, and minimum values of accuracies for MLR + CPMTPg were calculated. Moreover, genes selected via the CPMTPg were analyzed.

**Fig. 3:**
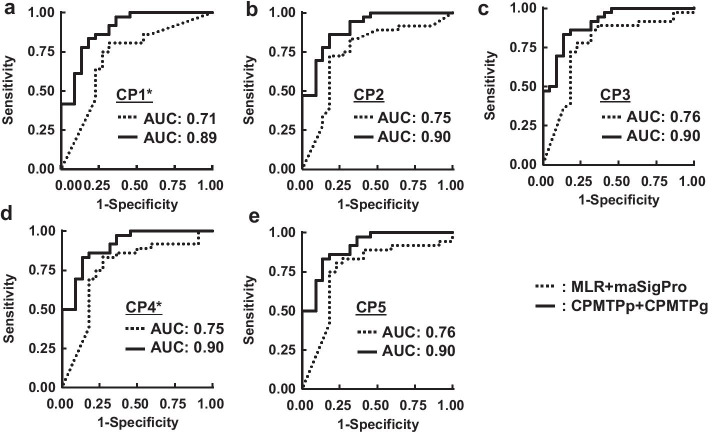
ROC curves of MLR + maSigPro versus CPMTPp + CPMTPg in HCV data; The CP of MLR + maSigPro presented two time points from “t-1” to “t.” The CP of CPMTPp + CPMTPg presented multiple time points from “t0” to “t.” “*” meant that the difference of AUCs between MLR + maSigPro and CPMTPp + CPMTPg was significant. **a** The case of CP1. **b** The case of CP2. **c** The case of CP3. **d** The case of CP4. **e** The case of CP5

The ROC curves and AUCs of MLR + maSigPro and CPMTPp + CPMTPg generated using the HCV dataset are shown (Fig. 3). Accordingly, the AUCs of MLR + maSigPro were 0.71 ($CP1$), 0.75 ($CP2$), 0.76 ($CP3$), 0.75 ($CP4$), and 0.76 ($CP5$), respectively. The AUCs of CPMTPp + CPMTPg were 0.89 ($CP1$), 0.90 ($CP2$), 0.90 ($CP3$), 0.90 ($CP4$), and 0.90 ($CP5$), respectively. The *p* values of the DeLong test were 0.03 ($CP1$), 0.06 ($CP2$), 0.07 ($CP3$), 0.05 ($CP4$), and 0.06 ($CP5$), respectively. The AUCs of CPMTPp + CPMTPg at all CPs were higher than the AUCs of MLR + maSigPro, and several time points showed a significant difference between these AUC values.

**Fig. 4:**
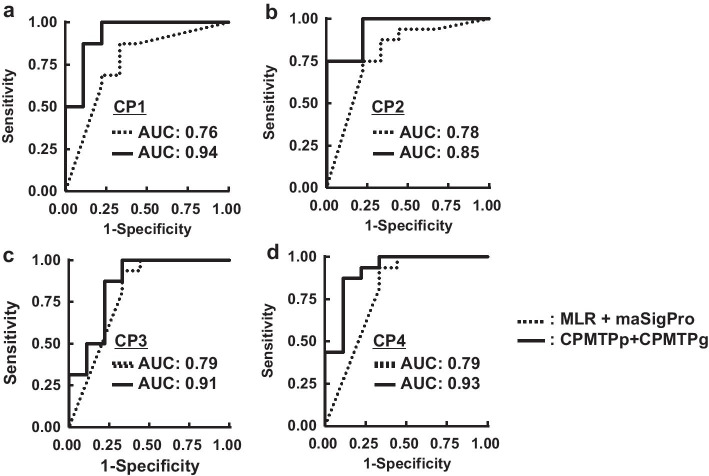
ROC curves of MLR + maSigPro versus CPMTPp + CPMTPg in MS data; The CP of MLR + maSigPro presented two time points from “t-1” to “t.” The CP of CPMTPp + CPMTPg presented multiple time points from “t0” to “t.” The difference of AUCs between MLR + maSigPro and CPMTPp + CPMTPg was not significant at all CPs. **a** The case of CP1. **b** The case of CP2. **c** The case of CP3. **d** The case of CP4

ROC curves and AUCs of MLR + maSigPro and CPMTPp + CPMTPg generated using the MS dataset are shown (Fig. 4). The AUCs of MLR + maSigPro from CP1 to CP4 were 0.76, 0.78, 0.79, and 0.79, while those of CPMTPp + CPMTPg were 0.94, 0.85, 0.91, and 0.93, respectively. The p-values of the DeLong test from CP1 to CP4 were 0.14, 0.68, 0.38, and 0.30. All AUCs of CPMTPp + CPMTPg were not significantly higher than those of MLR + maSigPro.

**Fig. 5:**
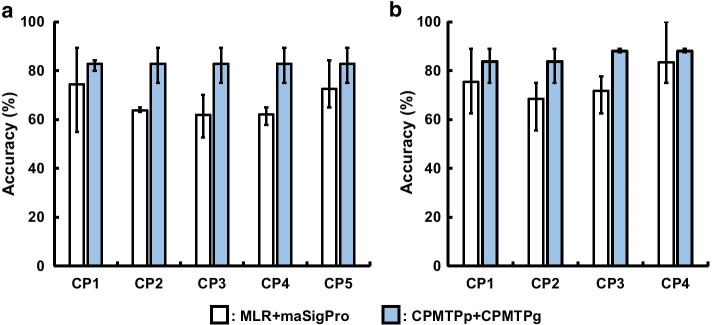
Accuracies of MLR + maSigPro versus CPMTPp + CPMTPg; The bars, top whisker, and bottom whisker were mean, maximum, and minimum values of accuracies by threefold cross-validation, respectively. **a** Using HCV dataset. **b** Using MS dataset

The accuracies calculated by MLR + maSigPro and CPMTPp + CPMTPg using the HCV dataset are shown (Fig. 5a). The mean accuracies of MLR + maSigPro from CP1 to CP5 were 74.4%, 63.7%, 61.9%, 62.0%, and 72.5%, respectively. The mean accuracies of the CPMTPp + CPMTPg were 82.8%, 82.8%, 82.8%, 82.8%, and 82.8%, respectively. The minimum and maximum values for the accuracies of MLR + maSigPro from CP1 to CP5 were 55.0% and 89.4%, 63.1% and 65.0%, 52.6% and 70.0%, 57.8% and 65.0%, and 65.0%, and 84.2%, respectively. The minimum and maximum accuracies of CPMTPp + CPMTPg from CP1 to CP5 were 80.0% and 84.2%, 75.0% and 89.4%, 75.0% and 89.4%, 75.0% and 89.4%, and 75.0% and 89.4%, respectively. The mean values of CPMTPp + CPMTPg were higher than those of MLR + maSigPro for all CPs. The maximum values for CPMTPp + CPMTPg, with the exception of CP1, were higher than those for MLR + maSigPro, while the minimum values at CPs were higher than those for MLR + maSigPro.

The accuracies of MLR + maSigPro and CPMTPp + CPMTPg for the MS dataset are shown (Fig. 5b). The mean accuracies of MLR + maSigPro from CP1 to CP4 were 75.4%, 68.5%, 71.7%, and 83.3%, respectively. The mean accuracies of CPMTPp + CPMTPg were 83.7%, 83.7%, 87.9%, and 87.9%, respectively. The minimum and maximum accuracies of MLR + maSigPro from CP1 to CP4 were 62.5% and 88.8%, 55.5% and 75.0%, 62.5% and 77.7%, and 75.0%, and 100.0%, respectively. The minimum and maximum accuracies of the CPMTPp + CPMTPg were 75.0% and 88.8%, 75.0% and 88.8%, 87.5%, and 88.8%, and 87.5% and 88.8%, respectively. The mean values of CPMTPp + CPMTPg were higher than those of MLR + maSigPro for all CPs. The CPs with maximum values for CPMTPp + CPMTPg that were higher than those of MLR + maSigPro were CP2 and CP3; however, the minimum values of CPMTPp + CPMTPg at all CPs were higher than those of MLR + maSigPro.

**Fig. 6:**
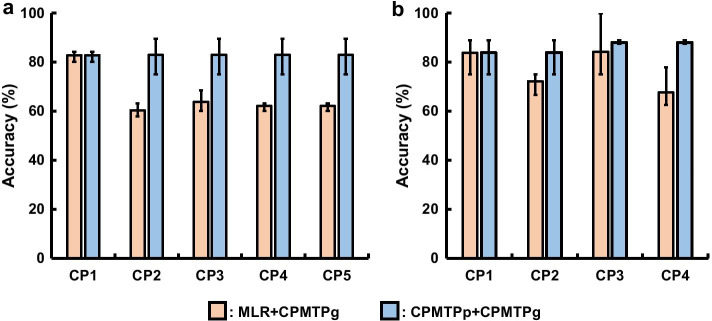
Accuracies of MLR + CPMTPg versus CPMTPp + CPMTPg; The bars, top whisker, and bottom whisker were mean, maximum, and minimum values of accuracies by threefold cross-validation, respectively. **a** Using HCV dataset. **b** Using MS dataset

The accuracies of MLR and CPMTPp estimated using the gene subset selected from the HCV dataset via CPMTPg are shown (Fig. 6a). The mean values of accuracies estimated by MLR + CPMTPg were 82.8 ($CP1$), 60.3 ($CP2$), 63.8 ($CP3$), 62.1 ($CP4$), and 62.1 ($CP5$), respectively. The minimum and maximum values of accuracies estimated by MLR + CPMTPg were 80.0% and 84.4% ($CP1$), 57.8% and 63.1% ($CP2$), 60.0% and 68.4% ($CP3$), 60.0% and 63.1% ($CP4$), and 60.0% and 63.1% ($CP5$), respectively. The mean, maximum, and minimum values of accuracies estimated by CPMTPp + CPMTPg were the same as those shown in Fig. 5a. The accuracy of MLR + CPMTPg at $\mathrm{CP}1$ was highest, while the accuracies for the other CPs decreased. On the other hand, the accuracy of CPMTPp + CPMTPg did not change with the increase in CPs.

MLR and CPMTPp were compared for accuracy using the MS subset (Fig. 6b). The gene subsets of MLR and CPMTPp were common. The mean values of the accuracies of MLR + CPMTPg were 83.7 ($CP1$), 72.2 ($CP2$), 84.2 ($CP3$), and 67.5 ($CP4$), respectively. The minimum and maximum values of accuracies of MLR + CPMTPg were 75.0% and 88.8% ($CP1$), 66.6% and 75.0% ($CP2$), 75.5% and 100.0% ($CP3$), and 62.5% and 77.7% ($CP4$), respectively. The mean, maximum, and minimum values of accuracies of CPMTPp + CPMTPg were the same as those shown in Fig. 5b. The accuracy of MLR + CPMTPg at “$CP1$” were different at each CP. The accuracy of CPMTPp + CPMTPg slightly improved as CPs increased.

The mean accuracies of CPMTPp + CPMTPg using the HCV dataset were not changed as time progressed (Fig. 6a). However, mean accuracies of the MS dataset improved slightly with increasing time (Fig. 6b). Further, the maximum and minimum values either did not change or improved slightly. Thus, in contrast to our hypothesis, the accuracies estimated using the two datasets either did not change or improved slightly with increasing time.

In the HCV dataset, 30 genes were selected by CPMTPg as the gene subset for the logistic regression model from the learning data on threefold cross-validation. The GO terms of the HCV dataset, which were determined by these genes, generated 4 clusters. The 10 GO terms had significant p-values (Table 4). “Repeat: 1”, “Repeat: 2” and “Repeat: 3”, which belonged to the same cluster and were selected by the same genes, were not terms associated with gene function. “Proteinaceous extracellular matrix,” “Disulfide bond,” and “Extracellular matrix” belonged to the same cluster, which was not the top cluster. “Disease mutation,” “Polymorphism,” “Visual perception,” and “Positive regulation of transcription, DNA-templated” did not belong to any cluster.

**Table 4:** Selected GO terms in HCV dataset

GO term	Genes	Count	*p* value	Cluster
Repeat:3	ADAM30, GFRA1, PSRC1	3	0.036	#1
Repeat:1	ADAM30, GFRA1, PSRC1	3	0.046	#1
Repeat:2	ADAM30, GFRA1, PSRC1	3	0.047	#1
Proteinaceous extracellular matrix	EFEMP1, WNT5A, KERA, OLFML2B	4	0.007	#2
Disulfide bond	ADAM3, EFEMP1, GFRA1, KLRC4-KLRK1, WNT5A, CACNA1A, IGLL1, KERA, OLFML2B, PRPH2	10	0.026	#2
Extracellular matrix	EFEMP1, WNT5A, KERA	3	0.045	#2
Disease mutation	EFEMP1, WNT5A, ACAT1, CACNA1A, CCND2, IGLL1, KERA, PRPH2, KCNK3, SRD5A2	10	0.039	Not belong
Polymorphism	AKAP5, ADAM30, EFEMP1, GFRA1, KLRC4-KLRK1, MAGEA10, ACAT1, CACNA1A, CAMTA1, CCND2, EIF3F, IGLL1, MED24, OLFML2B, OGDHL, PRPH2, PSRC1, PCDHGA3, SRD5A2, ZNF43, ZNF512B, ZNF711	22	0.030	Not belong
Visual perception	EFEMP1, KERA, PRPH2	3	0.036	Not belong
Positive regulation of transcription, DNA-templated	WNT5A, MED24, PSRC1, ZNF711	4	0.040	Not belong

These terms have lower *p* values than 0.05 (significance level). Thirty-one GO terms belong to four clusters. On the other hand, 13 GO terms do not belong. The clusters were generated during the GO analysis

Twenty-six genes were selected by CPMTPg using the MS dataset, where 4 were selected twice in threefold cross-validation. The GO terms of the MS dataset were decided according to these genes, and 3 clusters were constructed (Table 5). The GO terms with significant p-values are shown (Fig. 5). “Fatty acid metabolism” belonged to the cluster, while “Nucleus” and “protein binding” did not belong to any cluster.

**Table 5:** Selected GO terms for MS dataset

GO term	Genes	Count	*p* value	Cluster
Fatty acid metabolism	NDUFAB, ACAA2, ALOX15	3	0.008	#1
Nucleus	LARP6, RBM47, CENPO, ESRRA, MTDH, MORF4L1, PA2G4, RSL24D1, ZBED1, ZNF516, ZNF614	11	0.033	Not belong
Protein binding	NDUFAB1, ACAA2, ALOX15, CENPO, ESRRA, MTDH, MAT2A, MORF4L1, PA2G4, RSL24D1, SERPINA, TRPC3, TRPM8, ZBED1, ZNF614	15	0.047	Not belong

These terms have lower *p* values than 0.05 (significance level). Twenty-eight GO terms belong to three clusters. On the other hand, 21 GO terms do not belong. The clusters were generated during the GO analysis

## Discussion

AUCs and accuracies calculated using our proposed method (CPMTPp + CPMTPg) were compared with those calculated using the conventional method (MLR + maSigPro) via threefold cross-validation. The results of both AUCs (Fig. 4) and accuracies (Fig. 5) suggested that our method could predict response to therapy accurately at multiple time points compared to the conventional method.

The AUCs of CPMTPp + CPMTPg were higher than those of MLR + maSigPro for all CPs in both the HCV and MS datasets (Figs. 3, 4). However, CPs that showed significant differences were “$CP1$” and “$CP4$” in the HCV dataset, while the differences in the MS dataset were not significant for any CP. This is due to the insufficient number of patients to perform the DeLong test, especially in the MS dataset, where the patient number was $25$. Almost all CPs did not show a significant difference; however, a common trend in both HCV and MS datasets was that the AUCs of CPMTPp + CPMTPg were higher than those of MLR + maSigPro at all CPs.

According to Fig. 5, the mean accuracies of CPMTPp + CPMTPg were higher than those of MLR + maSigPro at all CPs, an observation common to both datasets. Moreover, the mean accuracies of CPMTPp + CPMTPg at each CPs were higher than the “$72.4\%$” cited in the reference [3] using the same HCV dataset. In the MS dataset, the mean accuracies of CPMTPp + CPMTPg at all CPs were also higher than the “$78.0\%$” cited in the reference [15].

In addition, the accuracies of CPMTPp + CPMTPg were confirmed for the artificial data. The results are shown in Additional file 4: Figure S4. The mean accuracies were more than 90.0% at all CPs.

CPMTPp was designed based on the hypothesis that more accurate prediction was dependent on data from more time points. However, the results of the comparison between MLR and CPMTPp (Fig. 6) did not support this hypothesis, although it indicated that CPMTPp continued to maintain accuracies as time points increased.

The accuracies of MLR, which did not consolidate the probabilities at multiple time points in the HCV and MS datasets, are shown (Fig. 6). In the HCV dataset, the top CP, which corresponded to the highest mean accuracy of MLR + CPMTPg, was “$CP1$”, after which the mean values corresponding to “$CP2$–$CP5$” decreased (Fig. 6a). The mean accuracies of MLR + CPMTPg for various CPs of the MS dataset appeared to be uncorrelated (Fig. 6b). The trends were also different regarding the maximum and minimum values. When the probabilities at multiple time points were not used for prediction as time points increased, the accuracies did not change or improve as in CPMTPp but were reduced or disjointed.

The above results indicated that prediction using more time points (CPMTPp) did not contribute to improved accuracy. However, MLR, which did not consolidate the probabilities of multiple time points, used the same subset of genes as CPMTPp, and its accuracy tended to decrease or fluctuate over time points. This trend was not changed by the gene selection method for maSigPro (Additional file 5: Figure S5). Therefore, it was found that the accuracies of CPMTPp contributed to maintaining accuracies as time points were processed, in contrast to MLR.

The gene subsets selected by CPMTPg were analyzed, and GO terms were extracted from the DAVID database (Tables 4, 5). Genes associated with terms that reportedly played an important role in diseases were discovered by reviewing previous studies that cited significant GO terms.

The GO terms (Table 4) included those that were reportedly associated with HCV infection. The extracellular matrix has been reported to develop progressive hepatic fibrosis and cirrhosis in 20% to 30% of HCV patients [38]. Previous studies have suggested that angiotensin II [38] and fibrogenic cytokines [39] contributed to the production of extracellular matrix in the liver. It was reported that excessive accumulation of extracellular matrix components, such as fibrillar type I and III collagens, fibronectin, and laminin, is a feature of liver fibrosis [40, 41]. Another study reported that the accumulation of extracellular matrix in liver fibrosis might impair the signaling of interferon used as therapy [40]. Regarding disulfide bonds, it was reported that a disulfide bond core protein complex might constitute the nucleocapsid-like particle of HCV [42].

The GO terms (Table 5) included those reported to be related to MS. It was suggested that “Fatty acid metabolism” may be a target for MS therapy since inhibition of carnitine palmitoyltransferase 1 (CPT-1), which is the rate-limiting enzyme in the beta-oxidation of fatty acids, contributes to a reduction in disease severity [43]. Especially, it was reported that when *ALOX15*, which encodes a fatty acid metabolizing enzyme, became functionally inactive, MS patients experienced more severe symptoms than when ALOX15 was active [44, 45].

The results of these numerical experiments using HCV and MS datasets suggested that CPMTP, our proposed method, may predict responses to therapy more accurately than the conventional method at multiple time points. Besides, CPMTP was able to select genes with functions associated with diseases from time-series microarray data.

CPMTP could be applied to gene expression data with arbitrarily selected multiple time points, and increasing time points did not affect the prediction model of CPMTP. CPMTP could be performed beyond the last time-point of treatment; however, it required validation. CPMTP could be applied to RNA-seq data and other gene expression data, which used a normalization similar to log2 fold-change and quantile normalization. When the proposed method is applied to the relatively large database, parameters and optimization methods, such as number of genes, number of samples, and number of time points, should be carefully considered.

## Conclusion

In individual patients showing specific therapeutic effects or occasional side effects, it is essential to accurately predict response to therapy using gene markers to determine a therapeutic strategy, such as changing or stopping therapy. Here, we propose a new prediction model and gene selection method termed CPMTP, which comprises a prediction component (CPMTPp) and selection component (CPMTPg). CPMTP was based on the hypothesis that more information related to time points provided a more accurate therapeutic response prediction. To enable CPMTPp incorporate more information from multiple time points, an overall probability of deciding a therapy response was estimated by consolidating the probabilities calculated at each time point, using the Bayesian theorem. CPMTPg selected the gene subset for use in the CPMTPp model via the optimization method, which was set as the fitness function of the consolidated probability.

CPMTP was evaluated using time-course gene expression profiles from HCV and MS patients in terms of accurate prediction, validation of the hypothesis, and gene function. These results suggested that CPMTP (CPMTPp + CPMTPg) predicted response to therapy accurately at all observed points compared to the conventional method. However, as opposed to our hypothesis, the predicted accuracy of CPMTPp was not improved but only retained as time points increased. Further, the gene subset selected by CPMTPg may be related to HCV and MS, according to analyses conducted by previous studies investigating the key GO terms associated with the gene subsets.

The above findings indicated that CPMTP might enhance long-term therapeutic procedures by accurately predicting response to therapy at multiple time points. Moreover, gene subsets identified by CPMTP may be useful as gene markers of disease progression. Thus, CPMTP may not only resolve difficulties associated with predicting response to therapy in HCV and MS patients but may also apply to the resolution of other clinical issues of a similar nature.

## Supplementary Information

**Additional file 1: Figure S1.** The pseudo-code of CPMTPp. This code predicted a therapy response of a patient. ${{\varvec{x}}}^{\left({t}_{r}\right)}=({x}_{1}^{{(t}_{r})},\dots ,{x}_{l}^{{(t}_{r})}) (r=1,\dots ,R)$: gene expression levels collected by the patient at time point “${t}_{r}$”. “$l$” was the number of genes in the gene subset. ${{\varvec{w}}}^{({t}_{r})}={({w}_{1}^{\left({t}_{r}\right)},\dots ,{w}_{l}^{\left({t}_{r}\right)})}^{\top }$: wights of the logistic regression at time point “${t}_{r}$”. ${w}_{0}^{{t}_{r}}$ was a constant term. $\widehat{y}$ was the predicted “sensitive” or “not sensitive” of the patient.

**Additional file 2: Figure S2.** The pseudo-code of CPMTPg:step1. This code created a gene pool by the step1 of CPMTPg. ${{\varvec{X}}}^{({t}_{r})}=\left({{\varvec{x}}}_{1}^{\left({t}_{r}\right)},\dots ,{{\varvec{x}}}_{l}^{\left({t}_{r}\right)}\right); {{\varvec{x}}}_{j}^{\left({t}_{r}\right)}={\left({x}_{j}^{\left(1,{t}_{r}\right)},\dots ,{x}_{j}^{\left(N, {t}_{r}\right)}\right)}^{\top } (j=1,\dots ,p)$: gene expression levels of “$p$” genes × “$N$” subjects at time point “${t}_{r}$”. ${y}^{(i)} (i=1,\dots ,N)$: the therapy response of the ${i}^{th}$ patient. ${\lambda }^{(k)} (k=1,\dots ,K)$: the ${k}^{th}$ values of lambda in Elastic Net in Stability Selection. The ${\varvec{G}}$ was the gene pool having genes selected by step1 of CPMTPg.

**Additional file 3: Figure S3.** The pseudo-code of CPMTPg:step2. This code created a gene pool by the step1 of CPMTPg. ${{\varvec{X}}}^{({t}_{r})}=\left({{\varvec{x}}}_{1}^{\left({t}_{r}\right)},\dots ,{{\varvec{x}}}_{l}^{\left({t}_{r}\right)}\right); {{\varvec{x}}}_{j}^{\left({t}_{r}\right)}={\left({x}_{j}^{\left(1,{t}_{r}\right)},\dots ,{x}_{j}^{\left(N, {t}_{r}\right)}\right)}^{\top } (j=1,\dots ,p)$: gene expression levels of “$p$” genes × “$N$” subjects at time point “${t}_{r}$”. ${y}^{(i)} (i=1,\dots ,N)$: the therapy response of the ${i}^{th}$ patient. ${\lambda }^{(k)} (k=1,\dots ,K)$: the ${k}^{th}$ values of lambda in Elastic Net in Stability Selection. The ${\varvec{G}}$ was the gene pool having genes selected by step1 of CPMTPg. The ${{\varvec{G}}}_{subset}$ was the gene subset of CPMTPp.

**Additional file 4: Figure S4.** Results of CPMTPp + CPMTPg using artificial data. The artificial gene expression data (1000 genes × 40 subjects × 5 time points; “#” in this figure means “number”) was created. This data subjects were 20 sensitive and 20 not sensitive responders. Gene expression levels of “Gene1”, “Gene2”, and “Gene3” were created by adding noise following a normal distribution (center:0; standard deviation:0.5) to each baseline. The baseline of “Gene1” had the different rising/ falling trends of gene expression levels between sensitive and not sensitive responders at all time points, while the baseline of “Gene2” and “Gene3” had it at a part of time points. Gene expression levels of the other genes were created by uniform distribution (maximum:1; minimum:5). To evaluate CPMTPp + CPMTPg, the threefold cross-validation was performed using this artificial data. As a result, CPMTPg selected “Gene1” from all genes as the gene subsets at all validation. These mean accuracies were 92.8%(CP1:${t}_{0}\sim {t}_{1}$), 97.6%(CP2:${t}_{0}\sim {t}_{2}$), 100%(CP3:${t}_{0}\sim {t}_{3}$), and 100%(CP4:${t}_{0}\sim {t}_{4}$), respectively. The accuracy at the early term was higher than 90%, and this value increased along with the time progressing. Similar trends were observed using actual datasets in this paper.

**Additional file 5: Figure S5.** Accuracies of MLR + maSigPro versus CPMTPp + maSigPro. The bars, top whisker, and bottom whisker represent mean, maximum, and minimum values of accuracies by threefold cross-validation, respectively. **a** HCV dataset. **b** MS dataset.

## Data Availability

The datasets generated during and/or analyzed during the current study are available in the GEO repository, https://www.ncbi.nlm.nih.gov/geo/ (GSE7123 and GSE24427). The artificial data used in this paper are available from the corresponding author on reasonable request.

## Abbreviations

HCV: Hepatitis C virus
MS: Multiple sclerosis
ANOVA: Analysis of variance
CPMTP: Consolidating probabilities of multiple time points
CPMTPp: Consolidating probabilities of multiple time points of the prediction
CPMTPg: Consolidating probabilities of multiple time points of gene selection
