# Supplementary figures and images for: Bayesian approach for predicting responses to therapy from high-dimensional time-course gene expression profiles
Source: BMC Bioinformatics. 2021 Mar 18;22:132. doi: 10.1186/s12859-021-04052-4 (PMC7977599; doi:10.1186/s12859-021-04052-4)

## Slide 1
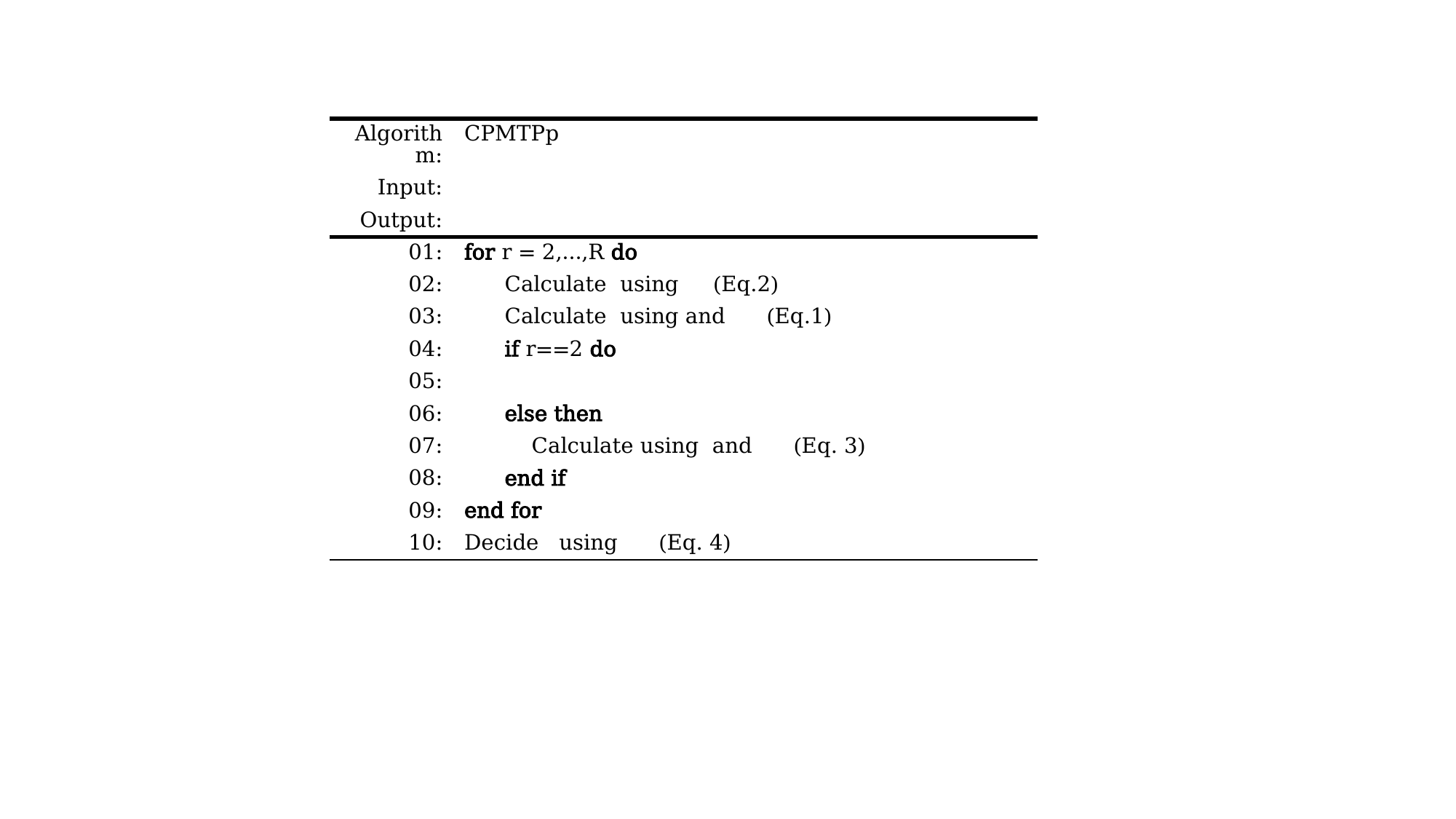

Supplement: Supplementary file 1 — Additional file 1: Figure S1. The pseudo-code of CPMTPp. This code predicted a therapy response of a patient. ${{\varvec{x}}}^{\left({t}_{r}\right)}=({x}_{1}^{{(t}_{r})},\dots ,{x}_{l}^{{(t}_{r})}) (r=1,\dots ,R)$xtr=(x1(tr),⋯,xl(tr))(r=1,⋯,R): gene expression levels collected by the patient at time point “${t}_{r}$tr”. “$l$l” was the number of genes in the gene subset. ${{\varvec{w}}}^{({t}_{r})}={({w}_{1}^{\left({t}_{r}\right)},\dots ,{w}_{l}^{\left({t}_{r}\right)})}^{\top }$w(tr)=(w1tr,⋯,wltr)⊤: wights of the logistic regression at time point “${t}_{r}$tr”. [file 12859_2021_4052_MOESM1_ESM.pptx]

## Slide 1
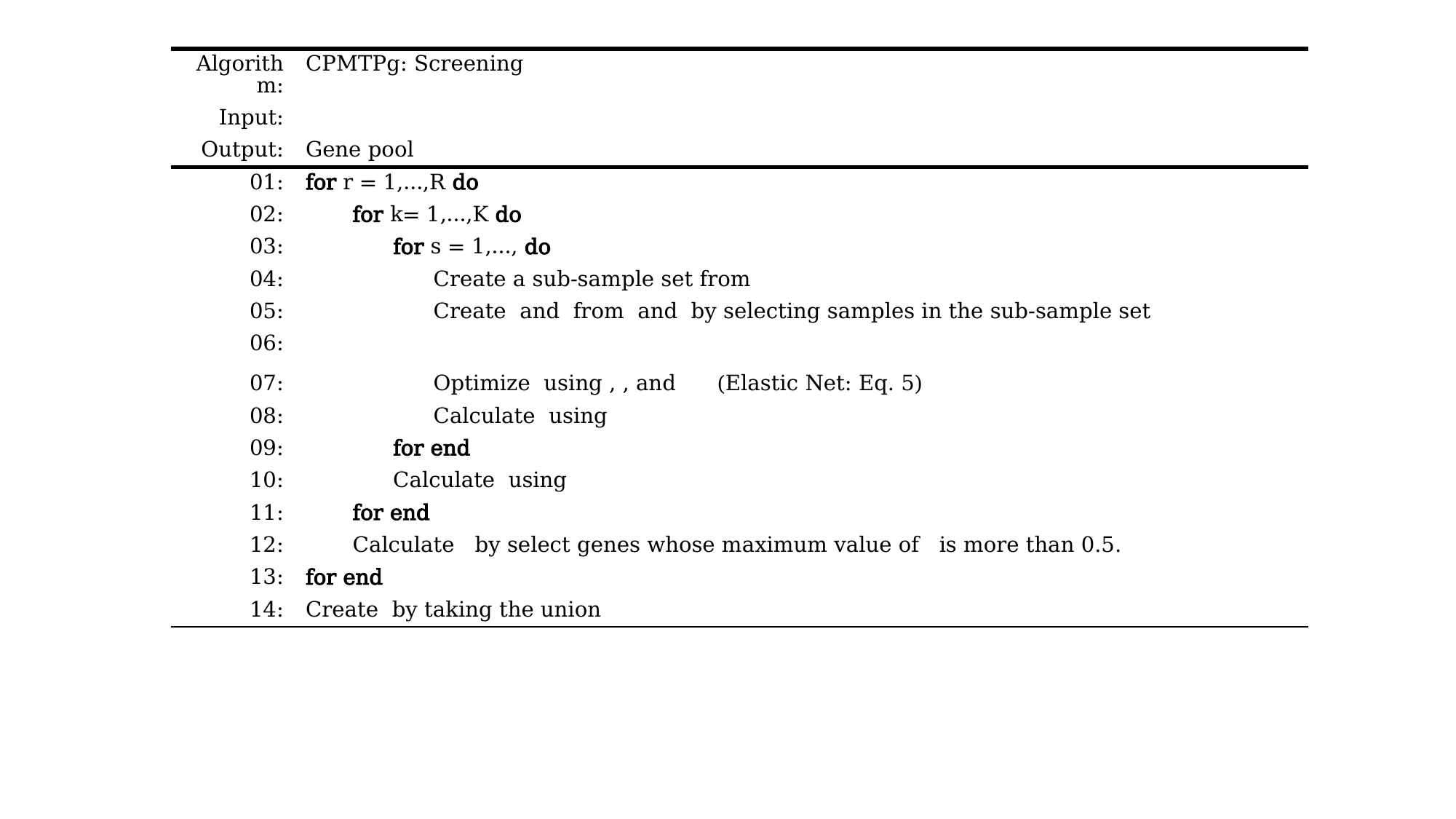

Supplement: Supplementary file 2 — Additional file 2: Figure S2. The pseudo-code of CPMTPg:step1. This code created a gene pool by the step1 of CPMTPg. ${{\varvec{X}}}^{({t}_{r})}=\left({{\varvec{x}}}_{1}^{\left({t}_{r}\right)},\dots ,{{\varvec{x}}}_{l}^{\left({t}_{r}\right)}\right); {{\varvec{x}}}_{j}^{\left({t}_{r}\right)}={\left({x}_{j}^{\left(1,{t}_{r}\right)},\dots ,{x}_{j}^{\left(N, {t}_{r}\right)}\right)}^{\top } (j=1,\dots ,p)$X(tr)=x1tr,⋯,xltr;xjtr=xj1,tr,⋯,xjN,tr⊤(j=1,⋯,p): gene expression levels of “$p$p” genes × “$N$N” subjects at time point “${t}_{r}$tr”. ${y}^{(i)} (i=1,\dots ,N)$y(i)(i=1,⋯,N): the therapy response of the [file 12859_2021_4052_MOESM2_ESM.pptx]

## Slide 1
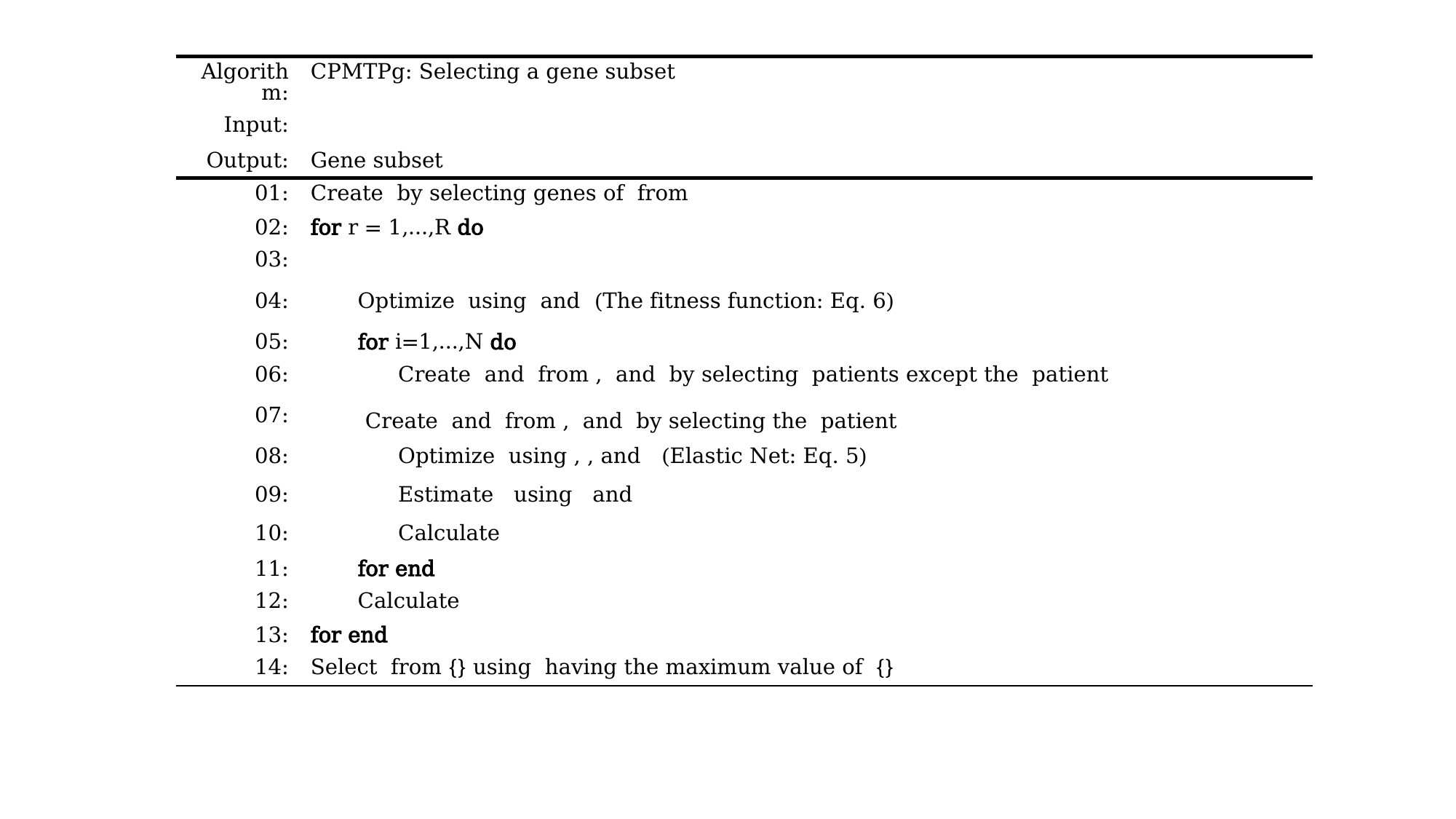

Supplement: Supplementary file 3 — Additional file 3: Figure S3. The pseudo-code of CPMTPg:step2. This code created a gene pool by the step1 of CPMTPg. ${{\varvec{X}}}^{({t}_{r})}=\left({{\varvec{x}}}_{1}^{\left({t}_{r}\right)},\dots ,{{\varvec{x}}}_{l}^{\left({t}_{r}\right)}\right); {{\varvec{x}}}_{j}^{\left({t}_{r}\right)}={\left({x}_{j}^{\left(1,{t}_{r}\right)},\dots ,{x}_{j}^{\left(N, {t}_{r}\right)}\right)}^{\top } (j=1,\dots ,p)$X(tr)=x1tr,⋯,xltr;xjtr=xj1,tr,⋯,xjN,tr⊤(j=1,⋯,p): gene expression levels of “$p$p” genes × “$N$N” subjects at time point “${t}_{r}$tr”. ${y}^{(i)} (i=1,\dots ,N)$y(i)(i=1,⋯,N): the therapy response of the [file 12859_2021_4052_MOESM3_ESM.pptx]
