# Supplementary material for: Bayesian approach for predicting responses to therapy from high-dimensional time-course gene expression profiles
Source: BMC Bioinformatics. 2021 Mar 18;22:132. doi: 10.1186/s12859-021-04052-4 (PMC7977599; doi:10.1186/s12859-021-04052-4)
Supplement: Supplementary file 4 — Additional file 4: Figure S4. Results of CPMTPp + CPMTPg using artificial data. The artificial gene expression data (1000 genes × 40 subjects × 5 time points; “#” in this figure means “number”) was created. This data subjects were 20 sensitive and 20 not sensitive responders. Gene expression levels of “Gene1”, “Gene2”, and “Gene3” were created by adding noise following a normal distribution (center:0; standard deviation:0.5) to each baseline. The baseline of “Gene1” had the different rising/ falling trends of gene expression levels between sensitive and not sensitive responders at all time points, while the baseline of “Gene2” and “Gene3” had it at a part of time points. Gene expression levels of the other genes were created by uniform distribution (maximum:1; minimum:5). To evaluate CPMTPp + CPMTPg, the threefold cross-validation was performed using this artificial data. As a result, CPMTPg selected “Gene1” from all genes as the gene subsets at all validation. These mean accuracies were 92.8%(CP1:${t}_{0}\sim {t}_{1}$t0∼t1), 97.6%(CP2:${t}_{0}\sim {t}_{2}$t0∼t2), 100%(CP3:${t}_{0}\sim {t}_{3}$t0∼t3), and 100%(CP4: [file 12859_2021_4052_MOESM4_ESM.pptx]

## Slide 1
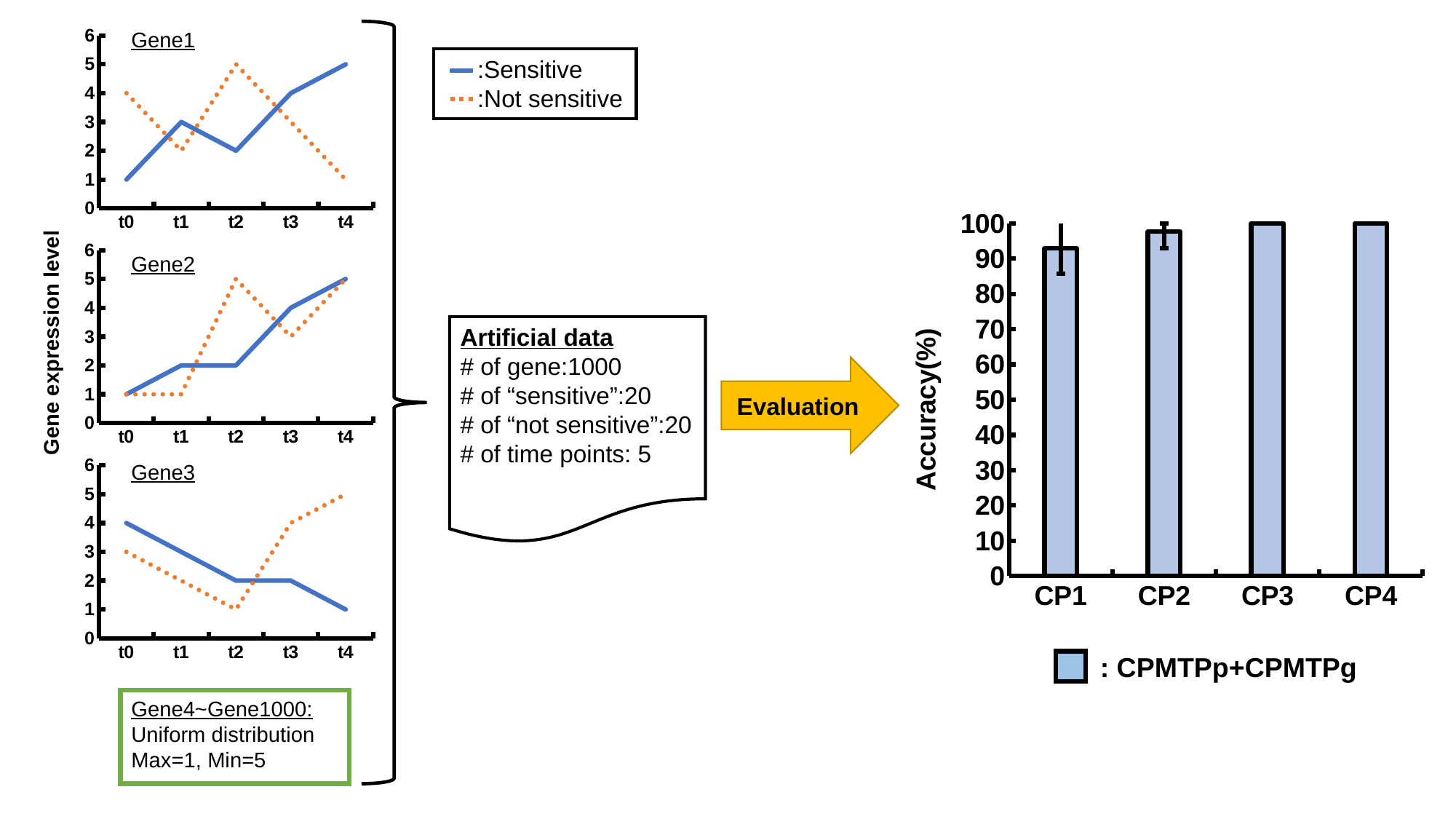

### Chart
| Category | Good | Poor |
|---|---|---|
| t0 | 1.0 | 4.0 |
| t1 | 3.0 | 2.0 |
| t2 | 2.0 | 5.0 |
| t3 | 4.0 | 3.0 |
| t4 | 5.0 | 1.0 |
:Sensitive
:Not sensitive
### Chart
| Category | Mean |
|---|---|
| CP1 | 92.85714285714285 |
| CP2 | 97.61904761904763 |
| CP3 | 100.0 |
| CP4 | 100.0 |
### Chart
| Category | Good | Poor |
|---|---|---|
| t0 | 1.0 | 1.0 |
| t1 | 2.0 | 1.0 |
| t2 | 2.0 | 5.0 |
| t3 | 4.0 | 3.0 |
| t4 | 5.0 | 5.0 |Gene expression level
Artificial data
# of gene:1000
# of “sensitive”:20
# of “not sensitive”:20
# of time points: 5
Evaluation
### Chart
| Category | Good | Poor |
|---|---|---|
| t0 | 4.0 | 3.0 |
| t1 | 3.0 | 2.0 |
| t2 | 2.0 | 1.0 |
| t3 | 2.0 | 4.0 |
| t4 | 1.0 | 5.0 |: CPMTPp+CPMTPg
Gene4~Gene1000:
Uniform distribution
Max=1, Min=5
