# Supplementary material for: Bayesian approach for predicting responses to therapy from high-dimensional time-course gene expression profiles
Source: BMC Bioinformatics. 2021 Mar 18;22:132. doi: 10.1186/s12859-021-04052-4 (PMC7977599; doi:10.1186/s12859-021-04052-4)
Supplement: Supplementary file 5 — Additional file 5: Figure S5. Accuracies of MLR + maSigPro versus CPMTPp + maSigPro. The bars, top whisker, and bottom whisker represent mean, maximum, and minimum values of accuracies by threefold cross-validation, respectively. a HCV dataset. b MS dataset. [file 12859_2021_4052_MOESM5_ESM.pptx]

## Slide 1
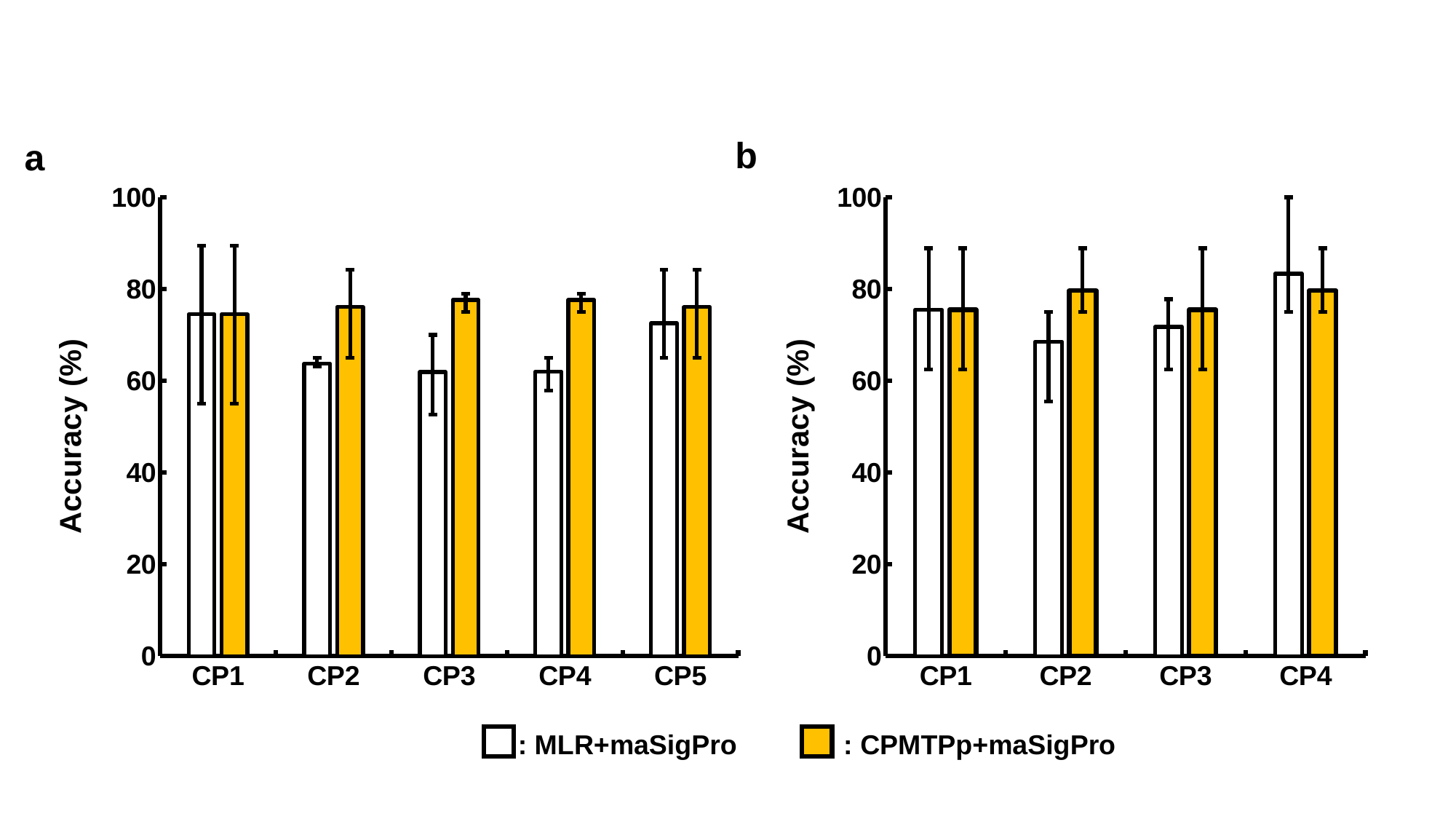

b
a
### Chart
| Category | maSigPro+MLR | maSigPro+OPM |
|---|---|---|
| CP1 | 74.4736842105263 | 74.4736842105263 |
| CP2 | 63.771929824561404 | 76.05263157894736 |
| CP3 | 61.92982456140351 | 77.6315789473684 |
| CP4 | 62.01754385964913 | 77.6315789473684 |
| CP5 | 72.5438596491228 | 76.05263157894736 |
### Chart
| Category | maSigPro+MLR | OLM+MLR |
|---|---|---|
| CP1 | 75.46296296296298 | 75.46296296296298 |
| CP2 | 68.51851851851853 | 79.62962962962963 |
| CP3 | 71.75925925925927 | 75.46296296296298 |
| CP4 | 83.33333333333333 | 79.62962962962963 |　: MLR+maSigPro : CPMTPp+maSigPro
